# HPV vaccination intention among guardians of male junior high school students in Chongqing, China

**DOI:** 10.3389/fpubh.2025.1692814

**Published:** 2025-11-17

**Authors:** Xiaoqin He, Ningpei Bai, Jiawei Xu, Ningyu Wan, Ying Xiao, Binyue Xu

**Affiliations:** 1 EPI Department, Chongqing Center for Disease Control and Prevention (Chongqing Academy of Preventive Medicine), Chongqing, China; 2 School of Public Health, Southwest Medical University, Luzhou, China

**Keywords:** HPV vaccine, vaccination willingness, vaccine hesitancy, male adolescents, guardianship

## Abstract

**Background:**

The human papillomavirus (HPV) vaccine is essential for preventing sexually transmitted infections and HPV-related cancers in both men and women. Vaccinating boys directly protects them and contributes to herd immunity. It also indirectly protects their future partners. As parents’ intentions strongly influence adolescents’ HPV vaccination, this study aimed to investigate the factors that influence their vaccination intentions for sons. The results of this study will provide a theoretical basis and practical recommendations for improving parental acceptance of HPV vaccination.

**Methods:**

A multi-stage, non-random, cross-sectional survey was conducted, targeting guardians of male junior high school students across various districts and counties in Chongqing.

**Results:**

A total of 3,481 valid responses were collected. Among the guardians, nearly 40% had never heard of HPV or the HPV vaccine. Overall, 71.3% of guardians expressed willingness to vaccinate their sons against HPV, while 28.7% were unwilling. Personal characteristics of the guardians, such as age and number of children, were associated with vaccination intention. Higher levels of knowledge about HPV were positively correlated with willingness to vaccinate, while vaccine hesitancy was associated with reduced acceptance.

**Conclusion:**

This study suggests that guardians in Chongqing have a high willingness to vaccinate their sons against HPV, and vaccine knowledge is one of the influencing factors. However, concerns about vaccine safety and efficacy remain significant barriers to vaccine acceptance. Awareness of the HPV vaccine should be improved, especially in groups with limited knowledge, through targeted educational campaigns and advice from healthcare professionals to address vaccine hesitancy.

## Introduction

Human papillomavirus (HPV) is the most prevalent sexually transmitted infection and is closely associated with genital warts as well as cancers of the cervix, anus, throat, and other sites (1). Although HPV is often considered a women’s health issue, men are equally susceptible (2) and play an important role in transmitting the virus to their partners, thereby increasing their risk of developing associated diseases (3–6). Vaccination is an effective strategy for preventing HPV infection and related diseases. The American Advisory Committee on Immunization Practices (ACIP) recommends HPV vaccination for women aged 13–26 and men aged 13–21 (7). As evidence of the vaccine’s benefits in disease prevention and herd immunity accumulates, many countries have begun including boys in their national vaccination programs (8, 9). In 2020, the World Health Organization launched the Global Strategy to Eliminate Cervical Cancer, proposing to increase the HPV vaccination rate among girls under 15 years old to 90% by 2030 (10). In China, several pilot programs to vaccinate adolescent girls have been implemented in recent years, including a subsidy program for schoolgirls launched by the Chongqing municipality in 2022 (11).

However, no equivalent program currently exists for boys, resulting in limited vaccine accessibility and suboptimal coverage among male adolescents. This gender disparity may hinder the overall effectiveness of HPV prevention strategies, given the important role of males in HPV transmission and their risk of developing HPV-related cancers (12). Therefore, expanding vaccination programs to include boys could yield significant public health benefits. Since the HPV vaccine is most effective before the initiation of sexual activity, and minors require parental or guardian consent for vaccination, it is important to understand guardians’ attitudes toward vaccinating boys.

This study aims to assess the willingness of the guardians of male junior high school students in Chongqing to vaccinate their sons against HPV and identify the factors influencing their decision. The findings will provide a scientific basis for improving vaccine acceptance and coverage among adolescent boys, as well as offering practical strategies.

## Methods

### Study design and participants

This study conducted a cross-sectional observational study on guardians of male junior high school students in Chongqing between February to May 2023.

The target population was parents or legal guardians of male junior high school students. A multistage, non-probability sampling method was used. To ensure feasibility, selection was made by local CDC staff based on factors such as geographic location, school size, and willingness to participate. At the time the study began, HPV vaccination for males had not yet been implemented in China. According to published studies, parental willingness to vaccinate their sons against HPV ranged from 41% to 87% (13). Thus, we conservatively assumed an expected acceptance rate of 50%. Taking into account a design effect of 2.5, stratified sampling, and an anticipated 20% non-response rate, the required sample size was calculated to be 2,400 parents.

### Ethics statement

This study was approved by the ethics committee of the Chongqing Center for Disease Control and Prevention (KY-2022-013). Informed consents were obtained from participants at the beginning of the survey.

### Questionnaire

A questionnaire was designed, and the socio-demographic information (such as age, number of children, residence, education, occupation, marital status, income, and relationship with children) of the respondents was collected as shown in Table 1. In addition, it covers knowledge about HPV and the HPV vaccine and the degree of vaccine hesitation. HPV and HPV vaccine awareness of vaccination was measured through a number of general knowledge questions. Vaccine hesitation was measured by the Vaccine Hesitation Scale (VHS), which was developed by the Strategic Advisory Expert Group (SAGE) Vaccine Hesitation Working Group (14). The main outcome was whether the guardian was willing to vaccinate the child against HPV. Options include “The child has been or is being vaccinated,” “willing to be vaccinated,” “unwilling to be vaccinated,” and “later.” With only one option to choose from, we combined the last two responses, both of which indicate no recent intention to get the HPV vaccine.

**Table 1:** Socio-demographic characteristics of guardians.

Guardians’ characteristics	Frequency (%)
Total	3,481 (100.0)
Age	
≤35 years old	494 (14.2)
36–40 years old	1,267 (36.4)
41–45 years old	739 (21.2)
>45 years old	981 (28.2)
Number of children	
One	1,037 (29.8)
Two	2,188 (62.9)
More than three	256 (7.3)
Gender of children	
Only boys	1,831 (52.6)
Male/female	1,650 (47.4)
Grade of children	
Junior Grade 1	1,325 (38.1)
Junior Grade 2	1,097 (31.5)
Junior Grade 3	1,059 (30.4)
Place of residence	
Rural	766 (22.0)
Urban	2,715 (78.0)
Marital status	
Married	3,085 (88.6)
Single (unmarried/divorced/widowed)	396 (11.4)
Annual household income	
Less than 100,000 yuan	2,377 (68.3)
More than 100,000 yuan	1,104 (31.7)
Relationship with children	
Father	900 (25.9)
Mother	2,499 (71.8)
Others	82 (2.3)
Educational level	
Junior high school or below diploma	1,685 (48.4)
High School or Technical School	1,089 (31.3)
College graduate or above degree	707 (20.3)
Occupation	
Production manufacturing industry	615 (17.7)
Agriculture	254 (7.3)
Business industry	859 (24.7)
Self-employed	827 (23.8)
Medical worker	66 (1.9)
Others	860 (24.6)
Have a relative with cervical cancer	
Yes	142 (4.1)
No	3,339 (95.9)
Communicate constantly with someone with a medical background	
Yes	1,244 (35.7)
No	2,237 (64.3)
Heard of cervical cancer	
Yes	3,055 (87.8)
No	426 (12.2)
Heard of the HPV	
Yes	2,330 (66.9)
No	1,151 (33.1)
Heard of the HPV vaccine	
Yes	2,173 (62.4)
No	1,308 (37.6)
District of school	
Rural	2,134 (61.3)
Urban	1,347 (38.7)
Age	
≤13 years old	1,353 (38.9)
14 years old	1,109 (31.9)
≥15 years old	1,019 (29.2)

Before making the questionnaire available to all participants, researchers surveyed a small portion of parents to ensure that it was easy to understand and complete. We conducted a reliability and validity analysis. Cronbach’s alpha coefficients are ≥0.6, indicating that the questionnaire is reliable.

### Data collection

The final survey was distributed online via the mini application ‘Wen Juan Xing’, a widely used online survey platform with functions equivalent to Amazon Mechanical Turk. Researchers commissioned the class teachers to send out the questionnaire in WeChat groups, the most commonly used means of communication among Chinese parents and teachers. The questionnaire began with informed consent, and all participants were allowed to proceed with the survey items only if they agreed to continue the survey. The data of 3,481 parents who completed the questionnaire were recorded in our study, while the other uncompleted questionnaires cannot be recorded on the platform. All the records were anonymous and without any personal identifiers.

### Statistical analysis

Descriptive statistics were calculated for sociodemographic characteristics, HPV and HPV vaccine awareness, and the number and percentage of vaccine hesitations. Knowledge scores (KS) were calculated to evaluate HPV and HPV vaccine knowledge, with one point assigned to each correct answer. Overall, KS were categorized into “low,” “medium,” and “high” levels. Vaccine hesitancy was assessed using a 10-item 5-point Likert scale, with responses ranging from 1 (strongly disagree) to 5 (strongly agree), resulting in a total score from 10 to 50. Based on the median, vaccine hesitancy was dichotomized into “low” and “high” for bivariate analyses. Willingness to vaccinate was assessed by the question: “Are you willing to get the HPV vaccine for your child right now?” Variables significantly associated with willingness to vaccinate in univariate analyses were included as covariates in the multivariable logistic regression model. Prior to fitting the model, multicollinearity among covariates was assessed using variance inflation factors (VIFs). All covariates had VIF values well below 5, indicating that multicollinearity was not a concern. Logistic regression was then used to identify factors independently associated with willingness to vaccinate, and results were reported as odds ratios (ORs) with 95% confidence intervals (CIs). Statistical significance was determined using two-tailed tests, with a significance level of 0.05. All analyses were conducted using R (version 4.4.2).

## Results

A total of 3,481 parents of male junior high school students were included in this study, of whom two children were the majority (62.9%), followed by only children (29.8%). Among the respondents, most are mothers (71.8%), have a junior high school education or below (48.4%), and have an annual household income of less than 100,000 yuan (68.3%).

### Characteristics of guardians

The characteristics of guardians and children are shown in Table 1. The average age of guardians was 41.8 ± 6.4 years old, and 78.0% of guardians lived in cities and towns. The majority (71.8%) were 45 years of age or younger, the majority (48.4%) had a junior high school diploma or less, and 20.3% had a university diploma or higher. In addition, 88.6% are married, 24.7% work as commercial or service workers, 23.8% are self-employed, and 17.7% work in manufacturing. Only 1.9% work in the medical field. A total of 68.3% of households have an annual income of less than 100,000 yuan. Most of the people who participated in the survey had two children. A total of 29.8% were only children.

### Awareness of HPV

**Figure 1:**
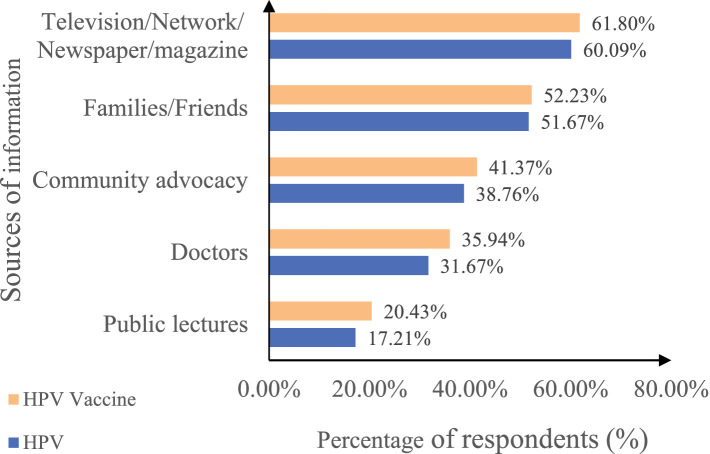
Main sources of information about HPV and HPV vaccine among guardians. The horizontal axis represents the percentage of respondents, and the vertical axis indicates the source of information. HPV, human papillomavirus; HPV vaccine, HPV vaccine. Different colors indicate the proportion of respondents who learned about HPV (blue) and HPV vaccine (orange) from each source. The questions were multiple choices in the survey.

Of the 3,481 guardians who participated in the survey, the vast majority (87.8%) had heard of cervical cancer, and 66.9% had heard of HPV. More than half learned about HPV through radio, television, or the Internet, and approximately half learned about HPV through introductions from family or friends (Figure 1). The study designed eight questions to assess guardians’ knowledge of HPV, with one point for each correct answer. Among guardians who had heard of HPV, the average score was 3.4 ± 2.2, with the lowest score being 0 and the highest score being 8. KS is divided into three grades: ‘low’ (less than 3 points), ‘medium’ (3–5 points), and ‘high’ (more than 5 points). Among guardians who had heard of HPV, 37.3% had low awareness of HPV, 44.9% had moderate awareness, and 17.8% had high awareness.

### Awareness of the HPV vaccine

In the survey, more than half (62.4%) of guardians had heard of the HPV vaccine. Of the guardians who had heard about the HPV vaccine, 61.8% learned about it through television, the Internet, newspapers, magazines, and 35.9% learned about it through doctors in hospitals or schools (Figure 1). We designed six questions to measure guardians’ awareness of the HPV vaccine, with one point awarded for each correct answer. Among guardians who had heard of the HPV vaccine, the average score was 3.5 ± 1.6, the lowest score was 0, and the highest score was 6. KS is divided into three grades: ‘low’ (less than 3 points), ‘medium’ (3–4 points), and ‘high’ (more than 4 points). Of the guardians who had heard about the HPV vaccine, 24.7% had a low level of cognitive knowledge about the HPV vaccine, 44.3% had a moderate level of cognitive knowledge about the HPV vaccine, and 31.0% had a high level of cognitive knowledge about the HPV vaccine.

### Degree of vaccine hesitancy

WHO’s SAGE Working Group on Immunization defined vaccine hesitancy as the act of delaying or refusing vaccines despite vaccine availability, and the VHS was developed to assess vaccine hesitancy (15). The VHS consists of 10 items that are rated on a 5-point Likert scale, ranging from ‘strongly disagree’ to ‘strongly agree.’ Among the 3,481 guardians, the average score was 22.4 ± 4.3, the lowest score was 10, and the highest score was 50.

Vaccine hesitancy was divided into two groups, with ‘low’ indicating ≤25 points and ‘high’ indicating more than 26 points. The sample median was used as the cutoff value. Among the guardians we surveyed, 78.7% had low vaccine hesitancy and 21.3% had high vaccine hesitancy.

### Attitude toward the HPV vaccine

A total of 71.3% of the guardians were willing to vaccinate their children against HPV, while only 28.7% were unwilling to vaccinate their children against HPV. Among them, 44.9% are worried that the child is too small and has an impact on its growth and development, 40.7% are worried about whether the vaccine really has a protective effect on the child after vaccination, 37.5% are worried about safety, and 81.2% are willing to vaccinate the child if the HPV vaccine is free of charge. Doctors’ and government recommendations for vaccination were the motivating factors for most of the respondents. A total of 54.9% did not know how to choose the vaccine, 20.8% chose the imported nine-valent vaccine, 15.6% chose the domestic two-valent vaccine, and 5.1% chose the imported four-valent vaccine.

### Factors associated with guardians’ willingness to vaccinate their children

Table 2 shows the results of the binary logistic regression analysis investigating the relationship between vaccination intention and various individual characteristics. The analysis revealed that guardians residing in rural areas were 1.268 (95% *CI*: 1.074–1.497, *p* = 0.005) times more likely to vaccinate their children with the HPV vaccine compared to those in urban areas. Guardians with both sons and daughters were 1.654 (95% *CI*: 1.346–2.031, *p* < 0.001) times more likely to vaccinate their children than those with only sons. Guardians who had vaccinated their children with all the free HPV vaccines were less willing to vaccinate their children than those who had not; the proportion was 0.574 (95% *CI*: 0.440–0.744, *p* < 0.001) times. Additionally, those who had heard of HPV were 1.418 (95% *CI:* 1.083–1.862, *p* = 0.01) times more likely to vaccinate their children than those who had not. Those with moderate HPV knowledge were 2.265 (95% *CI*: 1.739–2.953, *p* < 0.001) times more likely to vaccinate than those with low knowledge, and those with high knowledge were 2.683 (95% *CI*: 1.634–4.531, *p* < 0.001) times more likely. Guardians with high vaccine hesitancy were 0.193 (95%*CI*: 0.160–0.231, *p* < 0.001) times less likely than those with low vaccine hesitancy to vaccinate HPV vaccine for their children.

**Table 2:** Binary logistic regression analysis of attitude to the HPV vaccine to vaccinate their children among guardians.

Variables	*p*	OR (95% CI)
Place of residence		
Urban		ref.
Rural	0.005	1.268 (1.074, 1.497)
Gender of children		
Only boys		ref.
Male/female	<0.001	1.654 (1.346, 2.031)
Whether your child has received all free vaccinations		
No/Unclear		ref.
Yes	<0.001	0.574 (0.440, 0.744)
Annual household income		
More than 100,000 yuan		ref.
Less than 100,000 yuan	0.114	0.862 (0.717, 1.036)
Whether your child has been vaccinated at their own cost		
No		ref.
Yes	0.476	1.064 (0.897, 1.262)
Number of children		
One		ref.
Two	0.094	1.206 (0.969, 1.501)
More than three	0.562	1.125 (0.759, 1.681)
Heard of HPV		
No		ref.
Yes	0.011	1.418 (1.083, 1.862)
Heard of the HPV vaccine		
No		ref.
Yes	0.145	0.811 (0.611, 1.075)
The awareness of HPV		
Low		ref.
Moderate	0.006	1.369 (1.089, 1.721)
High	0.007	1.527 (1.127, 2.081)
The awareness of the HPV vaccine		
Low		ref.
Moderate	<0.001	2.265 (1.739, 2.953)
High	<0.001	2.683 (1.634, 4.531)
The degree of vaccine hesitancy		
Low		ref.
High	<0.001	0.193 (0.160, 0.231)

## Discussion

This study involved a cross-sectional survey in Chongqing, China, to investigate guardians’ willingness to have their sons vaccinated against HPV. The results showed that 71.3% of guardians were willing to vaccinate their sons, a proportion significantly higher than that reported among French parents of teenagers (16) and Greek guardians of children (17), and also higher than that among college students in China (18). In the United States and Canada, parental willingness ranges from 55% to 68% (19, 20), while in Spain, only approximately 24% of young Hispanic men believed the HPV vaccine could prevent future health problems (21). These cross-national differences likely reflect variations in vaccination policies, public health campaigns, and cultural or healthcare-related factors. The relatively high willingness observed in this study indicates that Chinese guardians—particularly in Chongqing—are receptive to HPV vaccination for boys when adequate information and policy support are provided. Strengthening health education and expanding HPV vaccination programs to include males could help translate this positive attitude into higher vaccination uptake.

Among the respondents, 36.4% of guardians were aged 36–40, an age group that pays particular attention to health issues. Studies indicate that the HPV infection rates are higher among men aged 26–35 (22), suggesting that guardians in this age group realize the importance of HPV vaccination and are therefore more willing to have their children vaccinated. This increase in willingness may be related to a deeper understanding of HPV-related diseases. Beyond age-related differences, rural guardians, despite generally having lower educational levels, were more willing to vaccinate their children than urban guardians. This finding aligns with research in Vietnam, where rural residents also demonstrated greater willingness to vaccinate (23). This pattern may reflect rural guardians’ perception of higher HPV risk and its potential consequences due to limited access to healthcare. Such heightened perception of severity and susceptibility could motivate stronger preventive behavior, thereby increasing their willingness to vaccinate sons.

In this study, 47.1% of guardians had some knowledge of HPV vaccines, higher than figures reported in systematic reviews and meta-analyses (37%) (24), and Nigeria (36.5%) (25), but lower than Romania (85.8%) (26), the United Kingdom (54.8%) (27), Kenya (48%) (28), and Thailand (60%) (29). Guardians familiar with HPV and HPV vaccines were more likely to choose vaccination. Those with a high level of knowledge were significantly more willing to vaccinate than those with lower knowledge, consistent with previous studies (30). This suggests that a better understanding of HPV and its link to cervical cancer leads to greater vaccine acceptance (31). Raising awareness of HPV and its vaccines among parents of junior high students, particularly those with low awareness, is therefore key to increasing vaccination rates.

Guardians with both sons and daughters were significantly more willing to vaccinate their children against HPV than those with only sons. Previous studies indicate that parents generally accept HPV vaccination for daughters more readily than for sons, and mothers are more inclined to vaccinate daughters (32). This reflects the perception that HPV vaccines are “female-only,” ignoring their protective effects for males against diseases such as anal and laryngeal cancers and condyloma acuminatum. HPV infection affects both males and females, and men are both at risk and important in transmission.

The quadrivalent and nine-valent HPV vaccines have been approved by China’s National Medical Products Administration (NMPA) for males aged 9 to 26, highlighting the country’s commitment to HPV prevention in men. Targeted health education should be strengthened to increase coverage. For example, Tianjin has integrated HPV vaccination into its public health program, providing subsidized vaccines for eligible females and promoting male vaccination following the nine-valent approval. Universities have also run campaigns to encourage a gender-neutral vaccination approach. Such efforts could serve as a model for other regions seeking to expand coverage across genders.

The survey found that vaccine hesitancy among guardians may prevent their sons from receiving HPV vaccines. In 2019, the World Health Organization (WHO) listed vaccine hesitancy as one of the “Top Ten Global Health Threats” and described it as “a potential serious challenge to immunization programmes.” The reasons given by guardians who participated in the survey for refusing HPV vaccines were generally concerns about the vaccine’s impact on children’s growth and development due to their young age, or doubts about its protective effect and safety. These findings are largely consistent with the results of similar research (33, 34). Overall, safety concerns are a key reason for HPV vaccine hesitancy (35, 36). Parents of vaccinated children stated that recommendations from doctors or healthcare authorities increased the likelihood of their children receiving the HPV vaccine. Doctors’ recommendations were an important factor in addressing vaccine hesitancy (37, 38). Similarly, in Spain, HPV vaccination among adolescent girls was strongly associated with advice from health professionals, highlighting that nurse and physician recommendations are key drivers of uptake (39). Promoting HPV vaccination should therefore involve clinical doctors through routine check-ups and vaccination clinics, which can reduce information barriers and enhance parental trust.

This survey revealed that 54.9% of guardians were uncertain about which vaccine to choose. Among recognized vaccine types, only 20.8% opted for the imported nine-valent vaccine, and 15.6% chose the domestic two-valent vaccine. This discrepancy between intention and choice may result from insufficient information, limited understanding of vaccine types, and concerns about cost and effectiveness. More systematic health education and information dissemination are recommended, with targeted publicity, particularly for urban and low-income families, to support informed decision-making. Improving information channels and providing professional consultation can enhance both vaccination intention and uptake, promoting wider HPV vaccine coverage.

This study has several limitations. First, its cross-sectional design allows only associations, not causal inferences. Second, the multistage non-probability sampling may introduce selection bias, though the sample was structured to reflect Chongqing’s demographic composition—a predominantly rural and mountainous region—ensuring reasonable representativeness. Third, while participants’ socioeconomic profiles generally matched census data, some subgroup imbalances may remain. Finally, self-reported information could be affected by socially desirable responses.

Despite these limitations, the study’s strengths include a structured and population-focused questionnaire, diverse guardian participation, and systematic assessment of key factors influencing male HPV vaccination willingness. Conducted before the official rollout of the male HPV vaccine in China, this research provides valuable real-world insights to support evidence-based policymaking and tailored vaccination education and promotion strategies in similar settings.

## Data Availability

The raw data supporting the conclusions of this article will be made available by the authors, without undue reservation.
